# Monolayer-Scale GaN/AlN Multiple Quantum Wells for High Power e-Beam Pumped UV-Emitters in the 240–270 nm Spectral Range

**DOI:** 10.3390/nano11102553

**Published:** 2021-09-29

**Authors:** Valentin Jmerik, Dmitrii Nechaev, Kseniya Orekhova, Nikita Prasolov, Vladimir Kozlovsky, Dmitry Sviridov, Mikhail Zverev, Nikita Gamov, Lars Grieger, Yixin Wang, Tao Wang, Xinqiang Wang, Sergey Ivanov

**Affiliations:** 1 Centre of Nanoheterostructure Physics, Ioffe Institute, 26 Politekhnicheskaya, 194021 St. Petersburg, Russia; nechayev@mail.ioffe.ru (D.N.); orekhova.kseniia@gmail.com (K.O.); nikpras@mail.ioffe.ru (N.P.); ivan@beam.ioffe.ru (S.I.); 2 Laboratory for Cathode Ray Pumped Lasers, P. N. Lebedev Physical Institute, Leninsky Ave. 53, 119991 Moscow, Russia; kozlovskiyvi@lebedev.ru (V.K.); dmidov@sci.lebedev.ru (D.S.); mzverev@mail.ru (M.Z.); 3 Department of Physics, Moscow Technological University, Vernadsky Ave. 78, 119454 Moscow, Russia; nikich-gam@yandex.ru; 4 Application Competence Center, Malvern Panalytical B.V., Lelyweg 1 (7602 EA), P.O. Box 13, 7600 AA Almelo, The Netherlands; lars.grieger@panalytical.com; 5 State Key Laboratory for Mesoscopic Physics and Frontiers Science Center for Nanooptoelectronics, School of Physics, Peking University, Beijing 100871, China; yxwang01@pku.edu.cn (Y.W.); wangshi@pku.edu.cn (X.W.); 6 Electron Microscopy Laboratory, School of Physics, Peking University, Beijing 100871, China; cwwangtao@pku.edu.cn

**Keywords:** monolayer thick GaN/AlN multiple quantum wells, III-nitrides, plasma-assisted beam epitaxy, electron-beam pumped ultraviolet-C emitters

## Abstract

Monolayer (ML)-scale GaN/AlN multiple quantum well (MQW) structures for electron-beam-pumped ultraviolet (UV) emitters are grown on *c*-sapphire substrates by using plasma-assisted molecular beam epitaxy under controllable metal-rich conditions, which provides the spiral growth of densely packed atomically smooth hillocks without metal droplets. These structures have ML-stepped terrace-like surface topology in the entire QW thickness range from 0.75–7 ML and absence of stress at the well thickness below 2 ML. Satisfactory quantum confinement and mitigating the quantum-confined Stark effect in the stress-free MQW structures enable one to achieve the relatively bright UV cathodoluminescence with a narrow-line (~15 nm) in the sub-250-nm spectral range. The structures with many QWs (up to 400) exhibit the output optical power of ~1 W at 240 nm, when pumped by a standard thermionic-cathode (LaB_6_) electron gun at an electron energy of 20 keV and a current of 65 mA. This power is increased up to 11.8 W at an average excitation energy of 5 µJ per pulse, generated by the electron gun with a ferroelectric plasma cathode at an electron-beam energy of 12.5 keV and a current of 450 mA.

## 1. Introduction

The (Al,Ga)N material system is of great importance for the development of semiconductor ultraviolet (UV) emitters used in compact mercury-free UV disinfection devices, spectroscopy, medicine, optical communication, etc. [1,2]. In particular, sub-250 nm UVC emitters have essential advantages for spectroscopic (Raman) substance detection and non-carcinogenic disinfection [3,4]. However, the achieved efficiency and output optical power of AlGaN-based UVC light-emitting diodes (LEDs) are still much lower than those of gas discharge Watt-range UV-lamps with a typical efficiency of 35% at 254 nm. Moreover, these parameters deteriorate significantly with the shortening of the emission wavelength, and currently, the best UV LEDs, emitting at 233 and 265 nm, exhibit the maximum emission power of 1.96 and 150 mW, respectively [5,6].

Most of the UV LEDs grown on commercially available *c*-sapphire substrates use AlGaN-based multiple quantum well (MQW) heterostructures with a high Al content above 50 mol% and a well width of 1.3–2.6 nm. They are characterized by a strong quantum-confined Stark effect (QCSE), which induces the spatial separation of charge carriers by built-in internal piezo- and pyroelectric fields, which reduces the effective bandgap and the radiative recombination rate [7,8]. An additional decrease in the output UVC power occurs with an increase in the Al content due to the transition from TE- to TM-dominant polarization mode of the output radiation, which has much lower extraction efficiency through the *c*-plane [9,10]. In addition, a higher Auger recombination and carrier leakage lead to a further deterioration in the efficiency of UV LEDs at high injection currents [11,12]. Finally, the achievement of high values of *p*- and *n*-type conductivity in Al-rich AlGaN layers should be called, perhaps, the most acute and difficult problem for UVB and UVC LEDs [13].

Among the various approaches to overcome the aforementioned problems, reducing the thickness of AlGaN QWs makes it possible to mitigate QSCE in the QWs, leading to a higher overlap of carrier wavefunctions [14]. Moreover, an increase in the difference between AlN mole fractions in the AlGaN-based QW and barrier layers also suppresses the TE–TM polarization switching due to the higher compressive stress in such QWs [15,16]. Ultimately, the use of AlGaN-delta-GaN QWs completely eliminates the polarization problem and drastically reduces the QCSE in the active regions of lasers [17]. Recent theoretical and experimental studies have shown that for an Al_0.61_Ga_0.39_N/AlN QW, a decrease in its thickness from 2 to 0.6 nm leads not only to a larger overlap integral of wave functions (up to ~0.65), but also provides a high exciton binding energy [18]. As a result, a 20-fold increase in the radiative recombination rate is observed in ultra-thin QWs.

The UV-emitting regions consisting of the ultrathin GaN QWs with a sub-monolayer (ML) thickness in AlGaN barriers were demonstrated by us in 2009 using the sub-monolayer digital alloying method developed for plasma-assisted molecular beam epitaxy (PA MBE) [19]. The high efficiency of radiative recombination in these ML-thick QWs heterostructures grown on *c*-sapphire substrates has been confirmed by observations of optically pumped UV-lasing and stimulated emission at room temperature (RT) in the spectral range of 258–303 nm with a minimum threshold optical power density of ~150 kW/cm^2^ [20,21]. Moreover, this approach has been applied to high-power UV-emitters pumped by an electron beam (e-beam), thus avoiding the aforementioned problem of *p*-doping of AlGaN layers with a high AlN content. As a result, such UV-emitters with pulse(cw) output powers up to ~160(39) mW at a wavelength of 285 nm have been demonstrated [22].

The possibility of tuning the effective bandgap from 3.4 to 6.0 eV with a decrease of the thickness of GaN QWs in the AlN matrix from several to one ML has been theoretically demonstrated in 2011 by Kamiya et al. [23] and experimentally confirmed by Taniyasu et al. [24] for the GaN/AlN superlattices grown by metalorganic vapor phase epitaxy (MOVPE). Then, ML-thick GaN/AlN-based MQW structures and UV-LEDs emitting within the sub-250 nm range were grown in Jena’s group using PA MBE [25,26,27,28]. In addition, e-beam pumped ML-GaN/AlN MQW UVC emitters grown by PA MBE and operated at 235 nm with the output optical powers in the continuous-wave (pulse-scanning) mode up to 28(150) mW were demonstrated by our group [29]. Similar MQW structures were grown by employing MOVPE by Wang et al. [30] and a maximum output power of ~2.2 W at 258 nm was achieved using a high-current pumping e-beam *I*_E_ = 37 mA. Recently, Toropov et al. [31] investigated the optical properties of ML-thick GaN/AlN single QWs, which, together with ab initio calculations, showed extreme 2D exciton confinement, being ideally suited for emission of UVC-light at 235 nm with an internal quantum yield as high as 75% at RT. An internal quantum efficiency of up to 50% was also demonstrated by Kawakami’s group for ML-thick GaN/AlN MQW structures emitting below 250 nm, which were grown using MOVPE [32]. We have recently published a review on such ML-thick GaN/AlN heterostructures [33].

In this work, we present results obtained using a set GaN/AlN MQW structures of an ML-scaled thickness grown using PA MBE under controllable metal-enriched conditions, which were used for both the well and the barrier layers. This leads to the realization of the spiral growth of dense, flat hillocks. This growth mode provides an atomically smooth ML-stepped terrace-like topology of the stress-free sub-2 ML-thick GaN/AlN MQW structures, which possess up to 400 wells and can emit in the spectrally narrow sub-250 nm UVC band with a pulsed output power in the several Watts range when pumped by an e-beam.

## 2. Materials and Methods

The AlN templates with a total thickness of 1.5–2 µm were grown by using PA MBE setup Compact 21T (Riber, Bezons, France) equipped by N_2_ plasma source HD-25 (Oxford Appl. Res. Ltd., Oxfordshire, UK) on standard *c*-Al_2_O_3_ substrates using migration-enhanced epitaxy for the 65-nm-thick nucleation layer and multi-stage metal-modulated epitaxy (MME) for the rest buffer layer at a substrate temperature varied between 780–850 °C [34,35,36]. These templates have an RMS roughness of about 0.6 nm and threading dislocation densities of ~5 × 10^9^ cm^−2^. *N* × {GaN_m_/AlN_n_} MQW structures (*N* = 25–400 the number of periods), *m* = 0.75–7 ML, *n* = 16 or 22 ML) were grown using PA MBE. The thicknesses are given in units of MLs, with the thickness of one ML being defined as halves of the corresponding *c*-lattice constants: 1ML_GaN(AlN)_ = 1/2*c*_GaN(AlN)_ = 0.259(0.249) nm [37]. Both GaN QWs and AlN barrier layers were grown under metal-enriched conditions using the Ga/N and Al/N flux ratios ~2 and ~1.1, respectively, at the same substrate temperature of 690 °C. This method has been described in detail previously [29]. All stages of PA MBE growth of AlN templates and ML-GaN/AlN MQW structures were monitored using a reflection high energy electron diffraction (RHEED) (Staib Instrumente GmbH, Langenbach, Germany), infrared pyrometer Mikron M680 (Mikron Infrared, Inc, Oakland, NJ, USA), as well as homemade laser reflectometry and multi-beam optical stress sensor [38]. The MQW structures were studied by using a high-resolution X-ray diffractometer (HRXRD) (Malvern Panalytical X’Pert^3^, Almelo, The Netherlands) with an X-ray wavelength of 0.15406 nm from Cu K_α_ radiation. The crystallinity of the structures was characterized using reciprocal space mapping (RSM) of around the (11–24) reflection and ω/2θ triple-axis scan of the symmetric (0002) reflection. The atomically resolved structure of the GaN/AlN MQWs has been analyzed by a high-angle annular dark-field scanning transmission electron microscopy (HAADF-STEM) using aberration-corrected Thermo Fisher Scientific Titan Cubed Themis G2 transmission electron microscope operated at 300 kV and equipped with a Bruker Super-X EDX detector. The cross-section specimens were prepared by conventional mechanical polishing followed by Ar+ ion milling.

Three types of e-guns were used for cathodoluminescence (CL) measurements of the samples. Initially, CL spectra were measured using an electron-probe microanalyzer Camebax (Cameca, Gennevilliers Cedex, France) equipped with an optical spectrometer, which covered the optical range from 200 to 470 nm [39]. Low-current CL spectra were measured with a typical beam energy of 10 keV and a continuous wave (cw) beam current *I*_e_ = 30 nA, whose diameter was 1 μm. For high-current measurements of the CL spectra, a homemade electron gun with a LaB_6_ cathode was used, which made it possible to obtain a 1.2-mm-diameter pulsed beam with an energy of up to 20 keV and a maximum current of 65 mA with a pulse duration of 100 ns and a frequency of 1.5 Hz. In this case, a temporary registration of the excitation current and intensity of the CL was performed. The third type of e-gun with a homemade ferroelectric plasma cathode provided the highest e-beam current of up to ~400 mA with a diameter of 4 mm at an e-beam energy varying from 5.6 to 12.5 keV. This e-gun provided pulsed e-beam excitation (3 µs, ~6 Hz).

## 3. Results and Discussion

### 3.1. Surface Topology of AlN Templates

**Figure 1:**
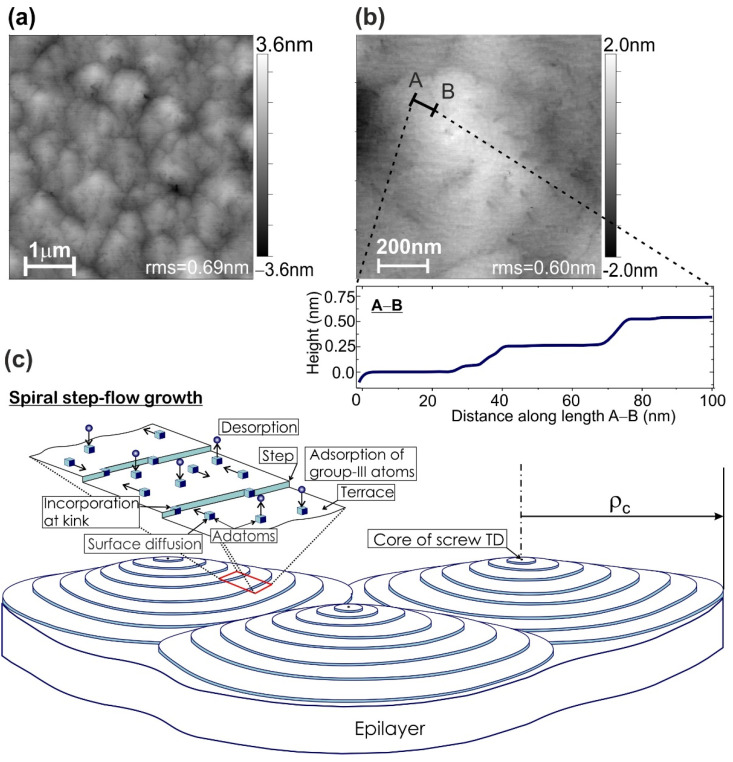
AFM images of the AlN buffer layer with a root mean square (rms) roughness of 0.69 and 0.60 nm, measured in the scanned areas of 5 × 5 µm^2^ (**a**) and 1 × 1 µm^2^ (**b**). Insertion shows the surface height profile measured along A–B length in (**b**). (**c**) Schematic diagram of spiral step-flow growth of III-Nitrides with inset illustrating the terrace–kink model of adatoms incorporation.

Figure 1a,b show the atomic force microscope (AFM) images of these templates, exhibiting all signs of their growth according to the spiral growth mechanism, which is typical for the growth of III-nitrides at the relatively high surface mobility of adatoms due to the use of metal modulated epitaxy (MME) under Me-rich growth conditions. The specific step-edge atomic bond configuration in wurtzite III-N films leads to the surface with stable ML-high steps [40]. The interaction of the advancing growth steps with numerous threading screw dislocations leads to the pinning of the steps at the intersection points of the dislocations with the surface and revolution of the growth front around the dislocation, resulting in the formation of the spiral growth hillocks [41,42,43]. Figure 1c illustrates this growth mechanism.

In accordance with the classical mechanism of step-flow growth described by Burton-Cabrera and Frank (BCF), the radius of curvature ($\rho_{c})$ of these hillocks is determined by the expression $\rho_{c}\sim 1/[kT\times ln(p/p_{0})]$, where T is the growth temperature, p and $p_{0}$ are the actual and equilibrium vapor pressures of the growth components, respectively [44]. In this expression, the denominator is the change in Gibbs free energy (−∆G)  per mole for a system going from pressure p  to $p_{0}$, and which is usually considered as the driving force for growth. The peculiarities of different growth mechanisms realizing in MOCVD and PA MBE environments are described by several groups [41,45,46].

One of the most distinctive features of PA MBE of III-Nitrides is the relatively low temperatures of epitaxial growth (690–850 °C for (Al,Ga)N alloys) that determines the strongly non-equilibrium growth environment due to a large difference between p  and $p_{0}$. As a result, this technology is characterized by the formation of spiral growth hillocks with relatively small radii. In the limiting case of very high deviations from equilibrium (i.e., at very high values of the *p*/*p*_0_ pressure ratio), a transition to the 2D-nucleation growth mechanism with infinitely small radii of growth grains is observed. Therefore, to enlarge the size of AlN growth grains—atomically smooth stepped hillocks with a size up to ~1 µm, the relatively high growth temperatures of about 800 °C and relatively low Al/N flux ratio were used in this work during MME growth of AlN buffer layers [36].

### 3.2. Surface Topology of GaN/AlN Heterostructures

**Figure 2:**
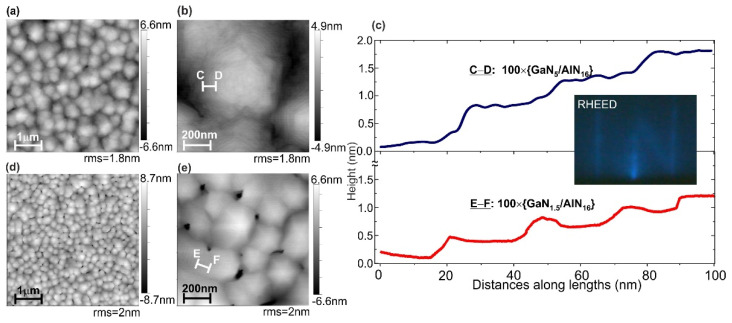
AFM images measured in scanned areas of 5 × 5 µm^2^ (**a**,**d**) and 1 × 1 µm^2^ (**b**,**e**) in the 100 × {GaN_5_/AlN_16_} (**a**,**b**) and 100 × {GaN_1.5_/AlN_16_} (**d**,**e**) MQW structures. (**c**) Blue and red lines indicate surface height profiles measured along C-D and E-F lengths in images (**b**) and (**e**), respectively. The inset shows a typical RHEED image observed during the growth of the MQW structures.

The *N* × {GaN_m_/AlN_n_} MQW structures were grown with the number of periods *N* = 100 and 400, consisting of GaN QWs and AlN barrier layers with thicknesses *m =* 0.75–7 MLs and *n =* 16 ML, respectively [29]. Figure 2a–e show the surface topology of two 100 × {GaN_m_/AlN_16_} MQW structures with *m* = 1.5 and 5 ML. Both structures with a thickness of about 500 nm exhibit the typical features of the spiral growth mode with the formation of tightly packed spiral hexagonal hillocks several hundred nanometers in diameter and steps with a height of about one *c*-crystallographic constant of ~0.5 nm (~2 ML). The terraces between the steps have a width of about 20 nm. During the growth of the MQW structures, the RHEED exhibited a streaky pattern (inset in Figure 2c), indicating a 2D morphology of the stepped hillock slope surface.

In general, the surface topology of the MQW structures is similar to the spiral hillock one observed during the PA MBE growth of AlN/*c*-Al_2_O_3_ templates described above. The increased density of the spirals in the MQW structures can be associated in accordance with BCF theory with a lower growth temperature of 690 °C leading to an increase in (*p*/*p*_0_) pressure ratio (supersaturation degree), as well as with relatively high Al and Ga excesses accumulated during growth of barriers and wells in the MQW structures. This assumption is also confirmed by observation in Figure 2a,d of the different hillock densities of 4 × 10^8^ and 2 × 10^9^ cm^−2^ for the structures grown using the different Al excess of 2.1 and 2.5 ML, respectively, which lead to the higher supersaturation in the latter.

**Figure 3:**
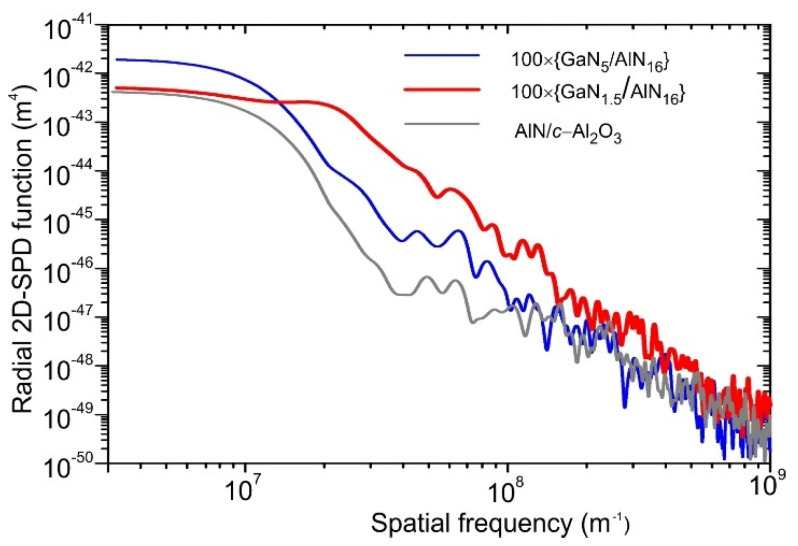
Radial 2D-spectral power densities as functions of surface frequency of the AlN template and MQW structures calculated from their AFM images measured in a scanned area of 1 × 1 µm^2^.

The observed grooves and pits between the flat hillocks determine the relatively high RMS roughness of about 2 nm over a large area of the structures. However, the local smoothness along the terrace surface is much better and its RMS roughness is significantly lower than the atomic step height (0.25 nm), as shown in Figure 2c. Moreover, Figure 3 shows the results of characterization of the surface topology of the AlN template and MQW structures by comparison of their radial 2D-spectral power density functions calculated from AFM images using Gwyddion software [47]. They indicate practically the same smoothness for all samples in the high surface frequencies (>10^8^ m^−1^). Some difference is observed only for low surface frequencies corresponding to larger irregularities related to the inter-grain boundaries in the MQW structures.

One should mention the preserving of the stepped morphology during the growth of rather thick MQW structures of 500 nm despite the possible stress (at *m* > 2 ML) and possible Ehrlich–Schwöbel barrier at the steps [48,49,50]. Indeed, the stress can induce step-bunching instability, described by Tersoff et al. [48] and Duport et al. [51], while the emergence of a barrier for downward diffusion can disturb the step-flow growth mode, leading to a local increase in the adatom nucleation and eventually transition to the 2D-island nucleation growth mode [52]; however, Kaufmann et al. [53] suggested that the formation of a group-III bilayer during PA MBE under metal-rich conditions makes it possible to screen Ehrlich–Schwöbel barrier; therefore, it can be assumed that the same effect was realized in our growth of both QWs and barriers under group-III rich conditions.

### 3.3. Stress Measurements

**Figure 4:**
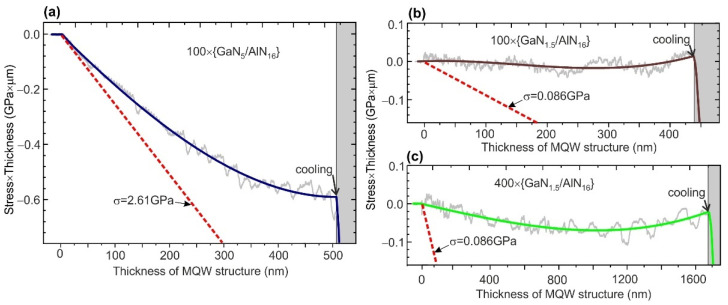
Evolutions of the products (stress × thickness) during the PA MBE of ML-thick *N* × {GaN_m_/AlN_n_} MQW structures with: (**a**) *m* = 5 ML, *n* = 16 ML, and *N* = 100; (**b**) *m* = 1.5 ML, *n* = 16 ML, and *N* = 100; (**c**) *m* = 1.5 ML, *n* = 16 ML, and *N* = 400. The dashed red lines in all plots show the calculated product (stress × thickness) with $\langle \sigma_{c}\rangle$ determined for the coherent growth of the studied heterostructures ${\mathrm{Al}}_{\langle x\rangle }{\mathrm{Ga}}_{1-\langle x\rangle }N/\mathrm{AlN},$ where 〈x〉=m/(m+n).

Figure 4a–c show the temporary evolutions of the measured products (average stress × thickness) (〈σ〉×h) during the growth of various GaN/AlN MQW structures on the relaxed AlN templates. In accordance with Stoney’s formula, these products are proportional to the substrate curvature [54,55]. In addition, the dashed red lines in each figure exhibit the calculated products ($\sigma_{c}\times h$), in which the values of stresses σ_c_ = *M*_AlGaN·_*ε* correspond to the coherent growth of the MQW structures with an average AlN content 〈x〉=m/(m+n) on the relaxed AlN layers with a biaxial modulus (*M*_AlGaN_) and strain (ε) calculated using the Vegard law.

MQW structure 100 × {GaN_5_/AlN_16_} exhibits a gradual relaxation of the compressive stress from the initial value corresponding to almost coherent growth to stress-free growth, when the thickness reaches about 500 nm, as shown in Figure 4a. On the contrary, Figure 4b,c demonstrate the curvature evolution during the growth of *N* × {GaN_1.5_/AlN_16_} MQW structures with *N* = 100 and 400, respectively, and indicate almost stress-free growth, beginning from the initial stage and continued throughout the whole growth runs. Such stress-free growth was observed by us previously in all MQW structures with the QW thickness below 2 ML [29].

This phenomenon can be explained by the stepped surface topology of GaN/AlN MQW structures. Indeed, this topology of the AlN barrier layer means its non-continuous filling by the ultra-thin GaN well layer. Consequently, the GaN/AlN lattice mismatch (−2.4%) can be elastically relaxed through the non-hexagonal strain mechanism, similar to the non-tetragonal strain relaxation in the materials with a cubic lattice [56,57,58,59]. This kind of stress relaxation has been described by Bourret et al. [60] for hexagonal ultrathin GaN/AlN heterostructures. The authors employed this mechanism to explain the observed zero stresses at the initial stages of the growth of ML-thick GaN platelets on AlN (and AlN platelets on GaN) using a low-temperature PA MBE under metal-rich conditions.

### 3.4. XRD Analysis

**Figure 5:**
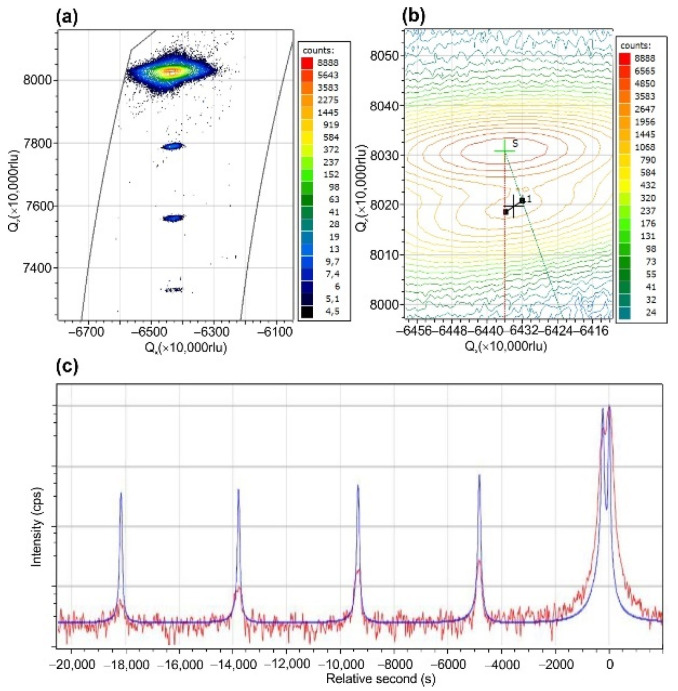
Results of high-resolution X-ray diffraction analysis of 100 × {GaN_1.5_/AlN_16_} MQW structure: (**a**,**b**) RSMs of asymmetric (11–24) peak plotted at different magnifications; (**c**) measured and simulated (0002) ω/2θ scans.

Figure 5a shows the RSM of 100 × {GaN_1.5_/AlN_16_} MQW structure with well-resolved MQW satellites aligned with a straight line at the same Q_x_ (in-plane reciprocal space vector) that indicates the same in-plane lattice parameter in this structure. A rigorous analysis of the RSM near the 0th MQW reflex, shown in Figure 5b, reveals its intermediate position between the vertical and inclined lines corresponding to the coherent and relaxed growths of the MQW structure on the stress-free AlN template, respectively. The position of this reflection corresponds to the degree of relaxation of 51% for this MQW structure with an average GaN content of 2.6 mol%. One should note that the XRD data on the MQW composition differ from the nominal average Ga content of 8.6% calculated as *m*/(*m* + *n*), although they do not correspond to the stress-free growth evaluated from the substrate curvature measurements during the growth (Figure 4b). The reasons for this discrepancy will be discussed below. Nevertheless, simulating the position of the satellite peaks in the ω/2θ scan of this MQW structure, shown in Figure 5c, yields an average period of 4.368 nm, which coincidences with the nominal MQW periodicity of 17.5 ML.

### 3.5. Study of GaN/AlN MQW Structures by HAADF STEM

**Figure 6:**
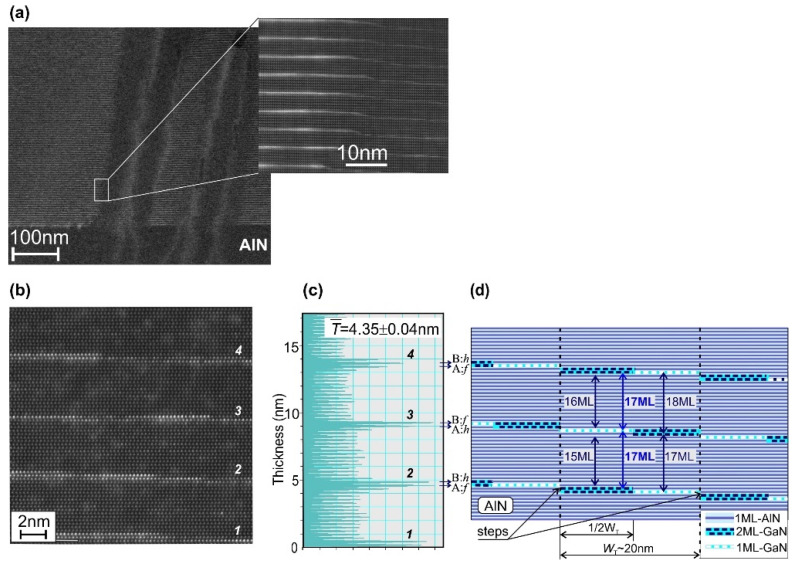
HAADF STEM images of *N* × {GaN_1.5_/AlN_16_} MQW structures with: *N* = 400 (**a**); 100 (**b**) taken at different magnifications. (**c**) Integrated distribution of brightness in image (**b**). (**d**) Schematic illustration of the stepped pattern of the MQW structure with *m* = 1.5 ML.

Figure 6a shows images of the 400 × {GaN_1.5_/AlN_16_} MQW structure obtained by a HAADF-STEM at low and high magnifications. The former was taken near the region of vertical depression with a significant disturbance of the crystallographic order of the MQW structure, which originates in the AlN template and is limited to a region with a diameter of about 100 nm. These bulk defects, apparently, can be associated with hollows on the structure surface, observed in the AFM images in Figure 2d,e. Importantly, outside these defect regions, a fairly uniform periodic distribution of dark AlN and bright GaN regions is observed (Figure 6a).

Figure 6b with HAADF-STEM image of the 100 × {GaN_1.5_/AlN_16_} MQW structure with nominally 1.5-ML-thick wells reveals their complex texture containing both 1- and 2-ML thick GaN QW regions. In general, this HAADF STEM image, together with Figure 6c, shows its integrated brightness distribution and corresponds to the stepped terrace-like topology of the structure surface observed in its AFM image shown in Figure 2c. Moreover, this distribution allowed us to determine the average MQW period of 4.35 ± 0.04 nm, which is very close to the nominal average period of 17.5 ML (4.36 nm).

Figure 6d shows a schematic sketch of the atomic layers in this MQW structure grown in the step-flow growth mode taking into account the fractional thickness of the wells *m* = 1.5 ML and nominally integer thicknesses of the barrier layers *n* = 16 ML. This sketch shows that even in the ideal step-flow growth, the fractionally thick MQWs have an imperfect distribution of one and two ML-thick QWs formed on the flat terrace-like AlN surfaces with an average width of about 20 nm. This imperfect periodicity, even for the nominal thickness of the QWs and barriers, can apparently be one of the reasons for the discrepancy between the nominal values and the average composition of MQW structures determined by XRD analysis, as described above; therefore, the MQW structure with a stepped morphology and imperfect periodicity should be considered as a weighted combination of ideal well–barrier pairs of different thicknesses, which are distributed along the (0001) growth direction and lateral directions, as suggested by Chandolu et al. [61] for the analysis of GaN/AlGaN SL with different types of imperfect periodicity.

In addition, some difficulties in the XRD analysis of the ultra-thin GaN/AlN MQW structures should be noted. For example, Gao et al. [62], using XRD ω/2θ scans of GaN_m_/AlN_n_ (*m* = 2–4 ML, *n* = 4–8 ML) SLs grown by MOVPE, determined for them somewhat smaller average *c*-constants in comparison with those calculated in accordance with the standard lattice constants. Some discrepancy between the average composition of the GaN_6_/AlN_18_ SLs, estimated from its RSM, and the nominal value of this parameter was observed by Enslin et al. in [63]. Apparently, all these discrepancies, including our results described in Section 3.4 can be related to the fact that the actual thickness of a single ML of GaN in the AlN matrix is just slightly lower than *c*/2 calculated using the lattice constant for bulk GaN of 5.185 Å (2 ML). However, this issue is beyond the scope of this paper and will be studied separately.

### 3.6. Study of Cathodoluminescence of 2D-GaN/AlN MQW Structures 

#### 3.6.1. Cathodoluminescence Excited by Continuous-Wave Low-Current e-Beam

**Figure 7:**
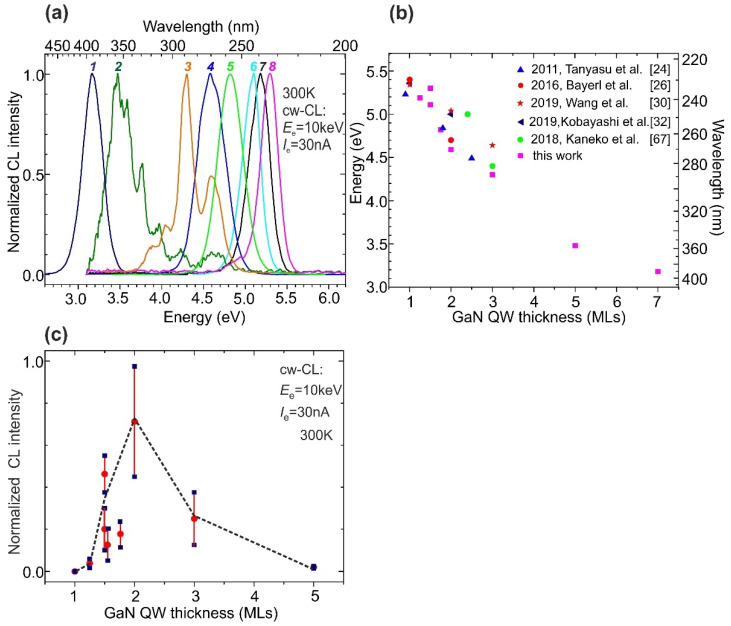
(**a**) Normalized CL spectra at RT of *N* × {GaN_m_/AlN_n_} MQW structures excited with a “low-current” standard thermoionic e-gun at the same *I*_e_ = 30 nA and *E*_e_ = 10 keV. The structures were grown with the following parameters: *1*—*m* = 7 ML, *n* = 16 ML, *N* = 25, all samples *2–7* were grown with the same *n* = 16 ML and *N* = 100, whereas *m* varied as *2*—*m* = 5 ML, *3*—*m* = 3 ML, *4*—*m* = 2 ML, *5*—*m* = 1.75 ML, *6*—*m* = 1.5 ML, *7*—*m* = 1.25 ML, in *8*—*m* = 1.5 ML, *n* = 22 ML, and *N* = 120 [29]. (**b**) Energies of PL and CL peaks measured for ML-scale GaN/AlN MQW structures grown using both PA MBE and MOVPE. (**c**) The dependence of the CL peak intensity on the nominal well thickness (*m*) in the MQW structures with CL spectra shown in (**a**).

Figure 7a shows CL spectra at RT of a series of 100 × {GaN_m_/AlN_16_}MQW structures with *m* varying from 1.25 to 7 ML and a total thickness of about 500 nm, which approximately corresponds to the penetration depth of an e-beam with an energy of 10 keV [64]. The thickness of AlN barriers was thick enough to exclude overlapping of the electron–hole wave functions from well to well, which would lead to the modification of the effective bandgap of the MQW structures [64]. 

The CL spectra *1–4* in Figure 7a show that a decrease in the nominal QW thickness from 7 to 2 ML led to a monotonic increase in the energy of single CL peaks from 3.0 eV (400 nm) to 4.6 eV (270 nm), respectively. This behavior is fully consistent with the theoretical calculations of the band diagram of GaN/AlN [23,65,66]. Such behavior is explained by both an increase in the confinement potential and a weakening of the QCSE in ultra-thin GaN/AlN QWs. Further reduction in the nominal fractional QW thickness from 1.75 to 1.25 ML continues to increase the CL peak energy up to 5.3 eV (235 nm), as shown by spectra *5–8* in Figure 7a. Figure 7b summarizes the experimental data on the positions of the photoluminescence (PL) and CL peaks of ML-scale GaN/AlN MQW structures grown in this work and by different groups using both PA MBE and MOVPE with different thicknesses of the GaN QWs [24,26,30,32,67].

Figure 7c shows a non-monotonic dependence of the integrated CL intensity on the QW thickness in the studied series of MQW structures. The initial increase in the CL intensity observed with a decrease in *m* from 7 to 2 ML corresponds in accordance with the first-principle calculations to mitigating the QCSE due to the suppressed spatial separation of charge carriers in the ultra-thin QWs. A further decrease in the intensity down to complete CL quenching at a nominal QW thickness of 1 ML or less (0.75 ML) cannot be explained by us at this stage of the study. The stepped structure of nominally flat QWs observed with a HAADF-STEM (Figure 6b) indicates that more in-depth studies are needed to describe radiative recombination in such structures, taking into account possible localization effects for carriers and excitons in the QWs with a varied thickness [25,31]. The MQW structures grown under various growth conditions will be investigated elsewhere by using comprehensive time-resolved measurements of their optical properties over wide ranges of temperatures, excitation type, power, etc.

Moreover, the observed narrowing of the full width at half maximum (FWHM) of the CL peak from ~380 to 234 meV (10 nm) with a decrease in the QW thickness from 5 to 1.5 ML, respectively, was unexpected. Indeed, the width of the CL peaks for the GaN/AlN MQW structures depends on random fluctuations of the well width and stress at nanoscale lateral lengths, which causes nanolocalized variations in the quantum confinement of charge carriers and the internal polarization field in the QWs. Both factors commonly lead to widening the CL peak with a thinning of the QWs [26]. In addition, broadening of the CL peak in the MQW structures with a total thickness of up to several microns can be caused by a change in the internal polarization field due to a gradual relaxation of stresses in the structures during their growth (Figure 4a and spectra *2, 3* in Figure 7a). In this case, the regions of MQW structures located at different depths emit CL with different energies. These considerations explain the relatively wide CL peaks observed in MQW structures with *m* > 2 and a thickness of ~2 µm, in which gradual relaxation of the compressive stress leads to different degrees of QCSE for CL emission from the different regions of the MQW structure. However, this stress non-uniformity is absent in the MQW structure with *m* < 2, confirming the stress-free growth, which means for these structures, constant compressive stress arises in all MQWs during post-growth cooling due to the difference in the thermal expansion coefficients.

#### 3.6.2. Cathodoluminescence Excited by Pulsed High-Current e-Beams

For the development of high-power e-beam pumped UV-emitters, two 360 (400) × {GaN_1.5_/AlN_22(16)_} MQW structures were grown in different growth series. A value of the well thickness was chosen as a compromise between a sufficient CL intensity and its rather short wavelength (<250 nm). The total thickness of structures of about 2 µm corresponds to the penetration depth of an e-beam with an energy of 20 keV. As will be shown below, these structures showed similar results, which characterizes the achieved run-to-run reproducibility of the growth method.

##### Thermoionic e-Gun with a LaB_6_ Cathode

**Figure 8:**
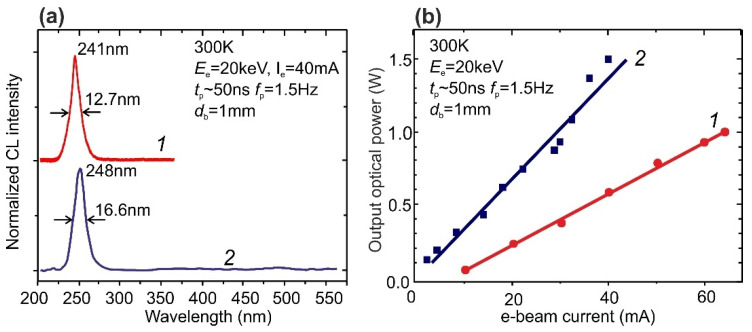
(**a**) Normalized CL spectra at RT of MQW structures with the similar nominal parameters *1*—360 × {GaN_1.5_/AlN_22_}, [29] and *2*—400 × {GaN_1.5_/AlN_16_} (this work). Both spectra were measured using a “high-current” pulsed e-gun with LaB_6_ cathode operated at the constant *E*_e_ = 20 keV and the *I*_e_ up to 65 mA with *t*_p_ ~ 50 ns and *f*_p_ ~ 1.5 Hz. (**b**) Output optical powers vs. the pulse-pumping currents for the (*1*) and (*2*) MQW structures in (**a**).

Initial high-current measurements of the MQW structures were carried out using pulsed an e-gun with a LaB_6_ cathode providing a maximum e-beam current of 65 mA. Figure 8a shows the CL spectra with single peaks at 241 and 248 nm for these nominally the same samples. One should note that the shapes and position of these spectra did not change with an increase in the pump current, and the maximum 1.5 Hz pulse output powers reached 1 W (at *I*_e_ = 65 mA, *E*_e_ = 20 keV) and 1.5 W (at *I*_e_ = 40 mA, *E*_e_ = 20 keV) for the MQW structures emitting at 241 and 248 nm, respectively, as shown in Figure 8b.

It is worth noting that the same e-beam LaB_6_ gun and measurement system was used in our study of the ML-thick GaN/AlN MQW structures grown using MOVPE [30]. Thus, comparable levels of the observed output powers of UV-emission in the spectral range of 240–260 nm and similar dependences of emission characteristics on QW parameters indicate the common nature of the observed phenomena in ML-scale MQW structures.

##### Cold e-Gun with a Ferroelectric Plasma Cathode

Finally, we investigated the CL spectra of the MQW structure using an ultra-high-power e-beam gun with a ferroelectric plasma cathode [68,69]. The main advantage of this type of e-gun with a cold discharge on a ferroelectric surface is the ability to achieve relatively high e-beam currents without overheating the cathode, which exists in the standard thermionic e-guns described above. On the other hand, this type of e-guns has poor reproducibility of the current pulses so far, which are a series of successive short pulses of different durations and amplitudes. 

**Figure 9:**
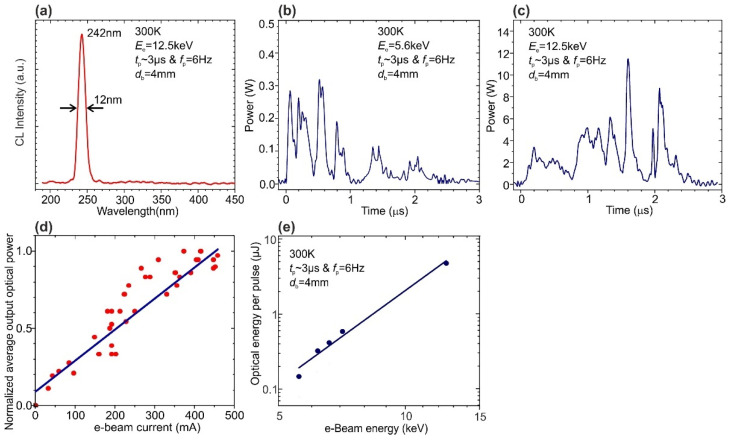
(**a**) Typical CL spectrum of the 400 × {GaN_1.5_/AlN_16_}MQW structure excited by an e-gun with a ferroelectric plasma cathode. Pulses of the output UV emission from this MQW structure excited by 4-mm-diameter e-beam with at *E*_e_ = 5.6 (**b**) and 12.5 keV (**c**) at pulse duration *t*_p_ ~ 2–3 µs and frequency *f*_p_ ~ 6 Hz. (**d**) Normalized average output optical power vs. e-beam current. (**e**) Dependence of the output UV optical energy per excitation pulse on the voltage applied to a ferroelectric plasma cathode of the e-gun.

Figure 9a exhibits a typical CL spectrum of the 400 × {GaN_1.5_/AlN_16_} structure with a single peak at 242 nm, the position, and shape of which did not change under various excitation conditions by an e-beam with a diameter of 4 mm emitted by an e-gun with a ferroelectric plasma cathode at various e-beam energy. It should be noted that the observed small difference (~6 nm) in the spectral positions of the CL spectra for the samples excited by electron guns with ferroelectric plasma or thermoionic LaB_6_ cathodes is associated with the fact that the samples under study were cleaved from different parts of the 2-inch substrate.

Figure 9b,c show typical pulses of the output optical power from this MQW structure, excited by an e-gun with ferroelectric plasma cathode providing e-beam with a pulse duration of about 3 µs and a frequency of ~6 Hz, while *E*_e_ could be varied from 5.6 to 12.5 keV, which led to a change in the maximum pulsed output optical power from 0.3 to 11.8 W, respectively. It is worth noting the absence of signature of the saturation effect on the dependence of the output optical power on e-beam current varied up to 450 mA, as shown in Figure 9d. By integrating the temporal variation of the output UV-emission power, the average output optical energies per pulse at different *E*_e_ were calculated. Figure 9e shows the dependence of this energy on *E*_e_, which reaches a maximum value of ~5 µJ/pulse at a maximum *E*_e_ = 12.5 keV.

## 4. Conclusions

We have demonstrated ML-scale *N* × {GaN_m_/AlN_n_} MQW structures (*N* = 100, 360, and 400, *m* = 0.75–7 ML, *n* = 16 or 22 ML) grown by using PA MBE on AlN/*c*-Al_2_O_3_ templates with employing the controllable metal-rich conditions. These structures exhibit signatures of a spiral growth mechanism of tightly packed flat hillocks with a diameter of about several hundred nanometers, which have a terrace-like stepped topology with a step height of ~2 ML and a width of about 20 nm. The stepped morphology of the QWs was confirmed by the HAADF STEM study of the MQW structures with a nominal QW thickness of 1.5 ML, while the XRD analysis revealed a satisfactory periodicity for these structures with the number of wells up to 400. Moreover, a stress-free growth of GaN_m_/AlN_n_ MQW was observed for the structures with ultra-thin QWs (*m* < 2 ML), which is explained by the elastic relaxation of stresses in such wells during their stepped growth, while the MQW structures with thicker wells (*m* ≥ 2 ML) exhibited the standard gradual relaxation of the compressive stress. Thinning the QWs from 7 to 1.25 ML led to a blue-shift of the wavelength of single-peak CL spectra from 400 to 235 nm, respectively, due to the increase in quantum confinement and mitigating QCSE in the wells.

It is important that the possibility of increasing the QW numbers up to 400 in 1.5 ML-thick GaN/AlN MQWs, due to their stress-free growth, allowed us to develop powerful e-beam pumped UVC emitters. Such emitters demonstrated the maximum UVC optical power of 1 and 1.5 W at wavelengths of 240 nm (half-widths of 11 nm) and 247 nm (16 nm), respectively, under pulsed excitation by an e-beam with *E*_e_ = 20 keV and *I*_e_ = 60 mA. Importantly that these emitters did not reveal the effect of power saturation with increasing the pump current. Finally, upon pulsed excitation of 400 × {GaN_1.5_/AlN_16_} MQW structure using a high-power electron gun based on a ferroelectric plasma cathode with an e-beam pulse duration and frequency of ~3 μs and 6 Hz, respectively, the output power of UVC emission at 242 nm could reach 11.8 W at *E*_e_ = 12.5 keV and *I*_e_ ~ 450 mA with an average radiation energy of 5 µJ per pulse.

## 5. Patent

Patent RU 2 709 999 C1 “Source of spontaneous ultraviolet radiation with wavelengths less than 250 nm” resulted from the work reported in this manuscript.

## Data Availability

The data presented in this study are available on request from the corresponding author.
